# Gastric sarcoidosis diagnosed with endoscopic ultrasound

**DOI:** 10.1002/ccr3.8623

**Published:** 2024-03-12

**Authors:** Husam El Sharu, Stephanie Ibarra, Ammad Chaudhary, Sinda Hidri, Zarak Khan, Danielle Hoo‐Fatt

**Affiliations:** ^1^ Department of Internal Medicine East Carolina University Greenville North Carolina USA; ^2^ Department of Internal Medicine Henry Ford Health System Detroit Michigan USA; ^3^ Department of Gastroenterology East Carolina University Greenville North Carolina USA

**Keywords:** endoscopic ultrasonography, gastric sarcoid, treatment outcome

## Abstract

**Key Clinical Message:**

Endoscopic ultrasonography (EUS) is crucial in diagnosing gastrointestinal sarcoidosis, especially when patients exhibit refractory abdominal symptoms. Our case highlights the significance of considering sarcoidosis in such cases and emphasizes the utility of EUS for accurate diagnosis and guiding appropriate treatment.

**Abstract:**

Gastrointestinal sarcoidosis is a rare and challenging manifestation of sarcoidosis that often presents with nonspecific abdominal symptoms, making diagnosis a complex process. We report the case of a 46‐year‐old African American female who experienced chronic epigastric abdominal pain, recurrent nausea, vomiting, and diarrhea for 15 years. Despite extensive investigations, including multiple biopsies, she was misdiagnosed with cyclic vomiting syndrome. Subsequently, an endoscopic ultrasound (EUS) revealed prominent lymph nodes and gastric granulomas, leading to a diagnosis of GS. This case underscores the importance of considering sarcoidosis in patients with refractory abdominal symptoms and highlights the utility of EUS in diagnosing this rare condition.

## 1 INTRODUCTION

Sarcoidosis is a multisystemic and autoimmune noncaseating granulomatous disease thought to affect African American females primarily. Although the systemic involvement with sarcoidosis is highly variable, in almost 90% of the cases, it involves the lungs and hilar lymph nodes, leading to interstitial fibrosis and lymphadenopathy, respectively.^1^ Less commonly involved organs include the skin, joints, eyes, heart, kidney, nervous, and digestive systems.^2^

In contrast to lung sarcoidosis, gastrointestinal (GI) tract sarcoidosis is rare, presenting in 0.1%–1.6% of cases, with the most common site of affection being the stomach, typically in the antrum.^1^, ^2^, ^3^ The most reported symptom is abdominal pain, but nausea, vomiting, anorexia, and early satiety were also reported.^4^

The manifestation of gastric sarcoidosis (GS) does not necessarily coincide with pulmonary sarcoidosis.^5^ The less specific and uncommon symptoms of nausea and vomiting of GI sarcoidosis further add to the complexity of the diagnosis.^4^ Therefore, in cases where GI sarcoidosis is suspected, endoscopic and histologic evaluations are deemed the most vital diagnostic procedures. Nevertheless, patients may have normal endoscopic features, and detecting granulomas with biopsies is complex, further complicating the diagnosis and requiring innovative methods for detecting such inflammation.^6^, ^7^ Herein, we report a case of gastrointestinal sarcoidosis diagnosed using endoscopic ultrasound (EUS) in a patient with multiple previous negative biopsies.

## 2 CASE PRESENTATION

A 46‐year‐old African American female presented to the hospital with a 15‐year history of chronic epigastric abdominal pain radiating to the back, intermittent, intractable nausea, non‐bloody vomiting, and diarrhea requiring recurrent hospital admissions without other complaints. Past medical history was pertinent for diet‐controlled type 2 diabetes mellitus and gallstone pancreatitis requiring cholecystectomy. Family history was significant for sarcoidosis in her brother. The patient smoked tobacco but denied using any recreational drugs, including marijuana. A physical examination was remarkable for severe diffuse abdominal tenderness. There were no palpable lymph nodes, skin manifestations, or abnormal lung sounds.

## 3 DIFFERENTIAL DIAGNOSIS AND WORKUP

Differential diagnoses include cyclic vomiting syndrome, infectious and immunological causes, along with sarcoidosis in the setting of family history.

Laboratory evaluation on multiple occasions showed normal liver function tests, lipase enzyme, thyroid‐stimulating hormone level, cortisol level, angiotensin‐converting enzyme level, and prolactin level. Infectious workups for human immunodeficiency virus, hepatitis viruses, clostridium difficile, stool parasites, and syphilis were unremarkable. Immunologic workup for celiac disease, paraneoplastic antibodies, and various rheumatologic conditions was unremarkable. Also, heavy metal screens and porphyrins were unremarkable.

Multiple computed topographies (CT) of the abdomen and pelvis were done along with magnetic resonance angiography to evaluate for vasculitis, but they were all unremarkable. Later in her disease course, a CT abdomen showed multiple new mild retroperitoneal lymphadenopathies in the celiac axis and porta hepatis.

The patient had antral erythema on multiple previous upper endoscopies, with biopsies revealing unexplained antral gastritis, and treated with prolonged courses of different proton pump inhibitors (PPI) without improvement. Furthermore, her previous colonoscopies were unremarkable. None of the previously done biopsies showed evidence of infectious processes assessed by staining. After all the extensive workup she had, she was diagnosed with cyclic vomiting syndrome.

Over the years, the patient tried sumatriptan, amitriptyline, Coenzyme Q, topimerate, levetiracetam, metoclopramide, dronabinol, olanzapine, and various other antiemetics with no relief.

**FIGURE 1:**
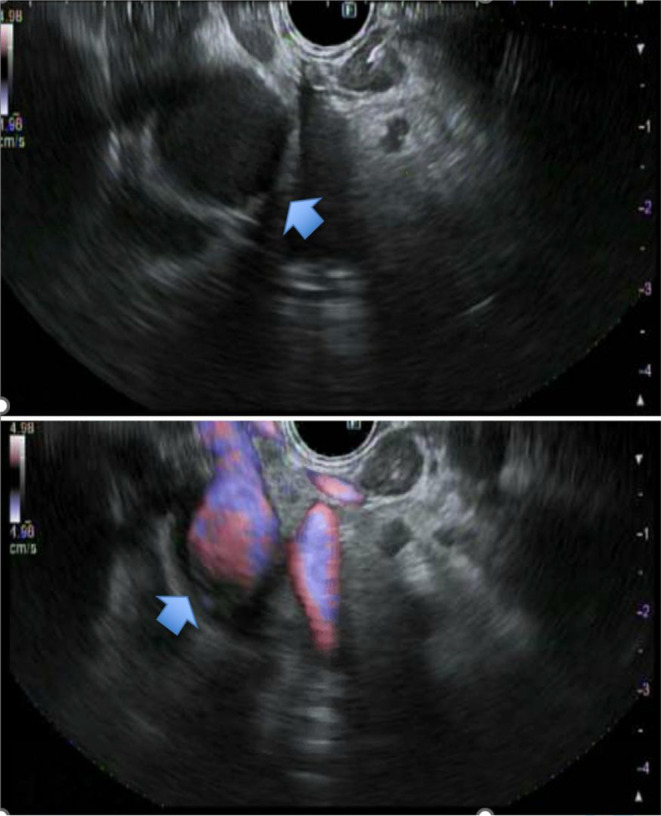
(A and B) shows an EUS with prominent lymph nodes in the perigastric and periduodenal region. (A) Shows the largest lymph node measuring 15 mm in maximal cross‐sectional diameter. (B) Shows doppler of the previously mentioned lymph nodes.

**FIGURE 2:**
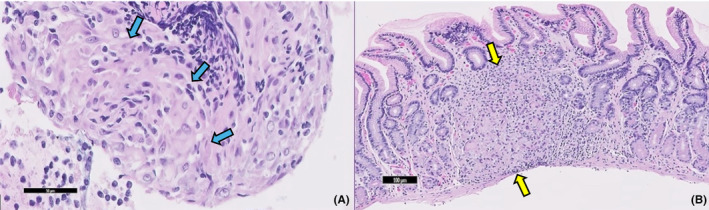
(A) High power photomicrograph (A, 200×) of hematoxylin and eosin‐stained section of cellblock preparation of a perigastric lymph node fine needle aspiration showing evidence of histocytes lining in a concentric fashion (Blue arrows) and (B, 100×) gastric biopsy demonstrating discrete collections of epithelioid histiocytes with abundant eosinophilic cytoplasm and indistinct cell borders, surrounded by a rim of inflammatory cells (Yellow arrows).

The patient had an endoscopic ultrasound (EUS) as a final resort. It showed a normal esophagus, stomach, and duodenum endoscopically. Ultrasonography showed a few prominent lymph nodes visualized in the perigastric and periduodenal region, with the largest measuring 15 mm in diameter, which was aspirated (Figure 1). Cytology obtained from the lymph node and gastric biopsy obtained from the opposite side of the lymph node showed noncaseating granulomas with negative Gomori methenamine silver and Acid‐Fast Bacillus stains (Figure 2). Following this, a chest CT was done, which was unremarkable. A CT head showed a juxtacortical hypodense area in the right frontal lobe, and a magnetic resonance imaging (MRI) head showed nonspecific single sub‐centimeter T2 FLAIR hyperintensity in the proper frontal white matter.

## 4 OUTCOMES AND FOLLOW‐UP

The patient was diagnosed with GS and started on prednisone 40 mg with significant improvement in symptoms. Initially, her symptoms were under control, temporarily resolving her episodic nausea and vomiting. Unfortunately, her symptoms recurred when her prednisone dose was tapered. Still, the patient refused to increase the prednisone dose again or to use steroid‐sparing agents, which resulted in the persistence of symptoms.

## 5 DISCUSSION

Sarcoidosis is an inflammatory disease manifested by noncaseating granulomas of unknown etiology that can invade any system.^1^ GI sarcoidosis can manifest as an extrapulmonary manifestation of systemic sarcoidosis, which can occur in 50%–60% of patients with pulmonary sarcoidosis but can occur as an isolated finding in 5%–9% of sarcoid patients.^5^ Typically, it is asymptomatic but can manifest with broad and nonspecific symptoms in 0.1%–0.9% of patients with systemic disease.^8^ The stomach, specifically the antrum, is the most common site of involvement of GI tract sarcoidosis.^9^

The differential diagnosis of GS is comprehensive and requires excluding various diseases, such as Crohn's disease, foreign body reaction, tuberculosis, histoplasmosis, Whipple's disease, and syphilis.^3^ The most significant limitations to diagnosing gastrointestinal sarcoidosis are its rare occurrence, broad symptomatology, and lack of specific testing, which may delay the initiation of appropriate treatment.^10^

The endoscopic findings in patients with GS include localized gastric infiltration, polyps, or possible ulceration. However, patients can also have a normal endoscopic appearance. If not suspected, multiple biopsies might be done before detecting the granuloma. Therefore, deep or full‐thickness biopsies are recommended to diagnose GS and to rule out other diseases.^6^, ^7^ Although not always present, sarcoidosis granulomas can be differentiated from Crohn's disease granulomas by the presence of Schaumann bodies, intracellular concentric calcifications, and prominent rather than sparse granulomas.^3^

More recently, EUS has become a well‐recognized tool for evaluating various GI diseases.^11^ However, the current diagnostic strategies for diagnosing GI sarcoid do not consider EUS. EUS is reliable for assessing mucosal thickening and better demonstrating the location of the disease process, thus aiding in determining the best site to biopsy and assess gastric and extragastric involvement, such as perigastric lymph nodes. Additionally, it can provide a measure for evaluating the resolution of the granulomatous inflammation in the post‐treatment phase.^12^, ^13^

Our patient underwent an extensive workup for broad symptoms, including endocrine, infectious, serologic, and toxicologic causes, which were grossly negative. Aside from a significant family history of sarcoidosis in her brother, there was no indication of sarcoid disease or intrapulmonary involvement at any point. Still, in addition to abdominal lymph nodes seen on one of the CT scans, multiple scattered hypodense lesions were seen on imaging in the liver and the brain, possibly dictating systemic disease. Due to these imaging findings, EUS was used to look for possible inflammatory and malignant etiologies.

Management of GI sarcoidosis is heterogeneous based on the severity of symptoms. Mild cases might be treated symptomatically with PPI. However, achieving symptom control with PPI only is unlikely; thus, adding 20–40 mg of prednisone is usually warranted with a regimen manifested as a single daily dose, with a gradual taper to a maintenance dose of 7.5–15 mg daily, guided by the clinical response over approximately 6 months.^14^, ^15^

Patients with chronic sarcoidosis usually require steroid‐sparing agents due to the long duration of treatment. In GI sarcoidosis, methotrexate is the most frequently used second‐line regimen due to its efficacy in symptom control over 24 months.^15^ One of the significant side effects of methotrexate is hepatotoxicity, causing an increase in transaminases and limiting their use, which has been thought to be related to the cumulative dose of the medication. Nevertheless, this must be interpreted cautiously, as sarcoid can also affect the liver. Baughman et al. have studied the effect of methotrexate on sarcoid patients, including liver sarcoidosis, and found a subtle increase in transaminases with the use of methotrexate, suggesting that the medication could still be used in patients with baseline elevated transaminases with no additional closer monitoring of transaminases.^16^

Owing to the disease's rarity, limited data quantify the rates of prognosis and recurrence of GI sarcoidosis. A study comprising a sample size of 25 patients with GI sarcoidosis demonstrated that up to 24% of the participants reported recurrent GI symptoms on follow‐up. Similarly, 44% of the patients reported ongoing extraintestinal symptoms, and 64% continued steroid therapy. Furthermore, it was stated that clinical remission of GI sarcoidosis occurred more frequently than Crohn's disease.^17^

## 6 CONCLUSION

Physicians should have a high index of suspicion of sarcoidosis in patients with multiple admissions with vague abdominal symptoms with grossly negative workup, inciting the use of EUS as a promising tool for evaluating granulomatous diseases.

## AUTHOR CONTRIBUTIONS

**Husam El Sharu:** Conceptualization; methodology; project administration; supervision; validation; visualization; writing – original draft; writing – review and editing. **Stephanie Ibarra:** Data curation; writing – original draft; writing – review and editing. **Ammad Chaudhary:** Methodology; writing – review and editing. **Sinda Hidri:** Methodology; writing – review and editing. **Zarak Khan:** Methodology; supervision; validation; visualization; writing – review and editing. **Danielle Hoo‐Fatt:** Supervision; validation; visualization.

## FUNDING INFORMATION

None.

## CONFLICT OF INTEREST STATEMENT

None.

## CONSENT

Written informed consent was obtained from the patient to publish this report in accordance with the journal's patient consent policy.

## Data Availability

All generated and analyzed data for this study are included in the manuscript.
